# Change of Petals′ Color and Chemical Components in *Oenothera* Flowers during Senescence

**DOI:** 10.3390/molecules23071698

**Published:** 2018-07-12

**Authors:** Yada Teppabut, Kin-ichi Oyama, Tadao Kondo, Kumi Yoshida

**Affiliations:** 1 Graduate School of Informatics, Nagoya University, Chikusa, Nagoya 464-8601, Japan; teppabut.yada@g.mbox.nagoya-u.ac.jp (Y.T.); kondot@info.human.nagoya-u.ac.jp (T.K.); 2 Research Institute for Materials Science, Nagoya University, Chikusa, Nagoya 464-8602, Japan; oyama@cic.nagoya-u.ac.jp

**Keywords:** cyanidin 3-*O*-glucoside, flower senescence, isosalipurposide, *Oenothera*, petal color change, quercitrin

## Abstract

*Oenothera* flower petals change color during senescence. When in full bloom, the flowers of *O. tetraptera* are white and those of *O. laciniata* and *O. stricta* are yellow. However, the colors change to pink and orange, respectively, when the petals fade. We analyzed the flavonoid components in these petals as a function of senescence using HPLC-DAD and LC-MS. In all three species, cyanidin 3-glucoside (Cy3G) was found in faded petals. The content of Cy3G increased in senescence. In full bloom (0 h), no Cy3G was detected in any of the petals. However, after 12 h, the content of Cy3G in *O. tetraptera* was 0.97 µmol/g fresh weight (FW) and the content of Cy3G in *O. laciniata* was 1.82 µmol/g FW. Together with anthocyanins, major flavonoid components in petals were identified. Quercitrin was detected in the petals of *O. tetraptera* and isosalipurposide was found in the petals of *O. laciniata* and *O. stricta*. The content of quercitrin did not change during senescence, but the content of isosalipurposide in *O. laciniata* increased from 3.4 µmol/g FW at 0 h to 4.8 µmol/g FW at 12 h. The color change in all three *Oenothera* flowers was confirmed to be due to the de novo biosynthesis of Cy3G.

## 1. Introduction

Flower color is an important characteristic for plants since it is related to pollination [1,2,3]. One of the many ways angiosperm plants attract pollinators is floral color changes [2,3]. Various mechanisms of color change have been reported such as changes in pH [4,5] and losses of pigment [6]. However, the most common physiological process is the appearance of a pigment especially an anthocyanin [2].

Anthocyanins provide the widest range of colors among the three major classes of flower pigments: anthocyanins, betalains, and carotenoids [7,8]. Many studies have explored the biosynthesis of these pigments [6,7,8,9,10,11]. In the case of anthocyanin, it is synthesized from phenylalanine, which is an amino acid, via a phenylpropanoid [10,11,12,13]. The pathway starts with the synthesis of naringenin chalcone from 4-coumaroyl-CoA and malonyl-CoA by chalcone synthase (CHS). Afterward, the chalcone is converted into flavanone, dihydroflavonol, and then leucoanthocyanidin [7,8,10,11]. Next, leucoanthocyanidin is oxidized and glycosylated to develop anthocyanin [7,8,10,11,14].

**Figure 1:**
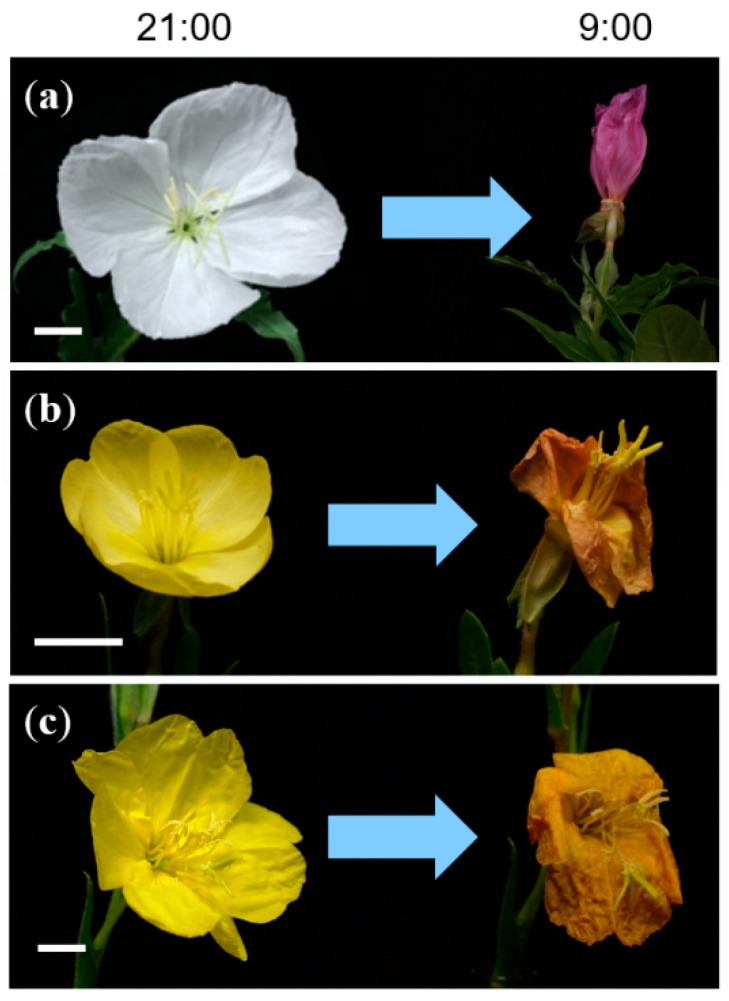
Flower color change in *Oenothera* petals during senescence. (**a**) *Oenothera tetraptera*, (**b**) *Oenothera laciniata*, and (**c**) *Oenothera stricta*. Scale bars: 1 cm.

A large number of plant taxa show floral color changes and one of them is genus *Oenothera*, evening primrose, which is known to undergo a flower color change during senescence. The flowers of this genus bloom in the evening and fade in the morning. When fully opened, the petals of *O. tetraptera* are white and then they become pink in the morning (Figure 1a). Those of *O. laciniata* as well as *O. stricta* are yellow and then they turn orange as they fade (Figure 1b,c). These phenomena strongly indicate that an anthocyanin is biosynthesized during senescence. However, the physiological process of color changes in *Oenothera* has not been confirmed. We investigated petal color change in these flowers and studied the mechanisms of such changes. Petal components were isolated and the constituents were identified. Afterward, the components were quantified according to the flower fading stage.

## 2. Results

### 2.1. Analysis of Petal Components of O. tetraptera

**Figure 2:**
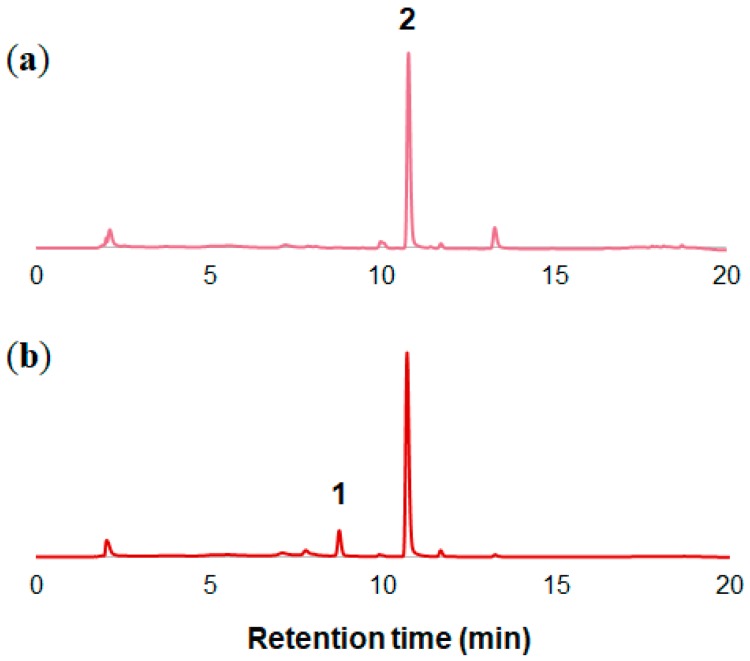
HPLC chromatograms of the extracts of the petals of *O. tetraptera*. (**a**) White petals at 0 h. (**b**) Pink petals at 12 h.

**Figure 3:**
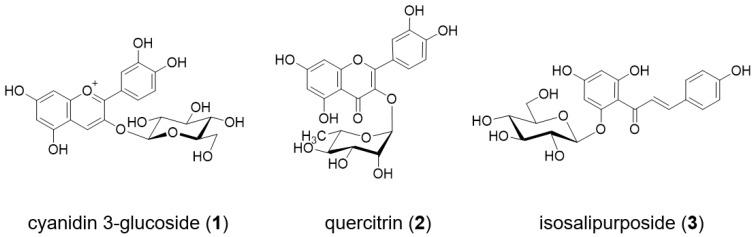
Chemical structure of the components of *Oenothera* petals.

As shown in Figure 1a, the petals of *O. tetraptera* bloom in white in the evening at approximately 21:00 and become pink after 12 h. To determine the chemical compounds responsible for the color change, the petals of *O. tetraptera* were collected at a full blooming white stage (0 h) and the faded stage (12 h). Then the petals were extracted with acidic solution (3% TFA in 50% CH_3_CN aq.). Each extract was analyzed by 3D-HPLC (Figure 2). In the white petals at 0 h, **2** was the major component. In the pink petals, which had undergone senescence, peak **1** appeared. Combined with the results of co-chromatography and the spectrum obtained from 3D-HPLC and LC-MS analysis (Figure S1) using an authentic sample, **1** was identified to be cyanidin 3-glucoside (Cy3G, Figure 3) [15]. Using the same procedure, **2** was determined to be quercitrin (quercetin 3-rhamnoside, Figure 3, and Figure S1). This result revealed that the red color change is due to the appearance of Cy3G during senescence.

**Figure 4:**
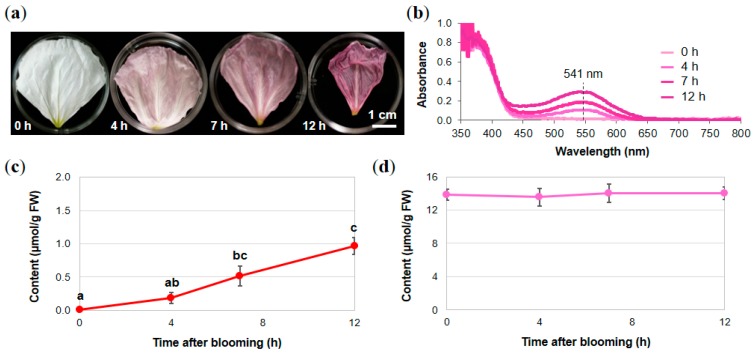
Changes in the color and flavonoid components of the petals of *O. tetraptera* during senescence. (**a**) Petal color at each stage, (**b**) reflection spectra, (**c**) change in the Cy3G (**1**) content, and (**d**) change in the quercitrin (**2**) content. The data displayed are the means ± SE of three replicates (*n* = 3). Where no error bars are shown, the SE was too small to determine. Different letters indicate significant differences according to Tukey’s HSD test (*p* < 0.05).

Since the components in *O. tetraptera* petals were identified, quantitative analysis of Cy3G (**1**) and quercitrin (**2**) during senescence was carried out. The petals at 0 h, 4 h, 7 h, and 12 h after blooming were collected (Figure 4a) and their reflection spectra were recorded (Figure 4b). The λ_vismax_ of the colored petals was 541 nm at each stage and the intensity at λ_vismax_ increased during flower development (Figure 4b). This corresponded with the L* value of the CIELAB color coordinate of the petals decreasing and the a* value increasing after blooming (Table 1). In addition, the pH of the pressed juice was measured and no obvious changes in pH were observed during senescence (Table 1). This indicates that the color change was not due to a pH change in the petals. When extraction from each petal was followed by HPLC analysis, the changes in the content of Cy3G (**1**) and quercitrin (**2**) were quantified (Figure 4c,d). The content of Cy3G increased during flower senescence and reached its highest level (0.97 µmol/g FW) 12 h after blooming (Figure 4c). In contrast, the content of quercitrin (**2**) at 0 h after blooming was 13.86 µmol/g FW, which is approximately 14 times more than the highest level of Cy3G. The content did not significantly change during senescence (Figure 4d).

### 2.2. Analysis of the Components of the Petals of O. laciniata and O. stricta

**Figure 5:**
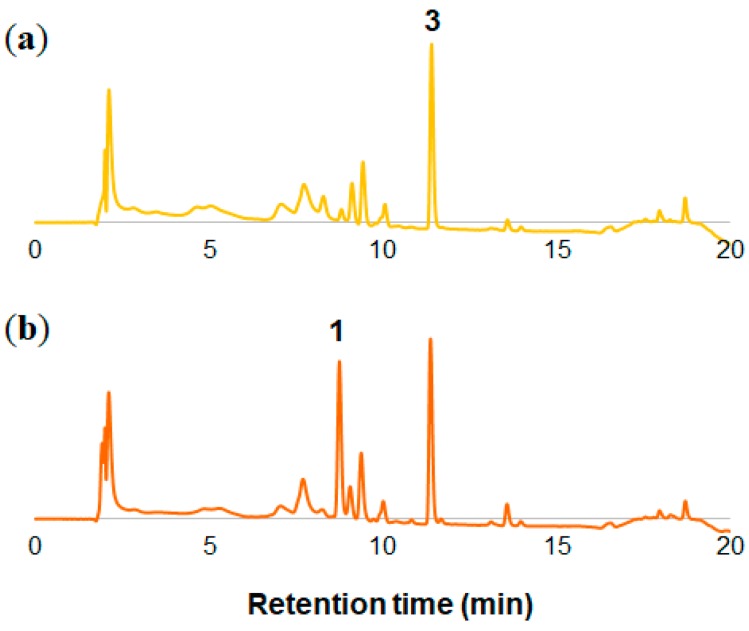
HPLC chromatograms of the extracts of the petals of *O. laciniata*. (**a**) Yellow petals at 0 h. (**b**) Orange petals at 12 h.

Next, the same experiments were done with *O. laciniata* and *O. stricta*. These flowers are yellow at full bloom and then turn orange during senescence (Figure 1b,c). The components of the petals of these two kinds of flowers were extracted and analyzed by 3D-HPLC (Figure 5 and Figure S2). As found in *O. tetraptera*, Cy3G (**1**) was detected at the senescence stage (Figure 5b and Figure S2b). For structure elucidation of peak **3**, the yellow petals of *O. laciniata* were extracted and peak **3** was isolated. Using MS (Figure S1) and NMR analysis (Figures S3–S8), **3** was identified to be isosalipurposide (chalconaringenin 2′-glucoside, **3**, Figure 3) [16,17]. The same compound was detected in petals of *O. stricta* (Figure S2).

**Figure 6:**
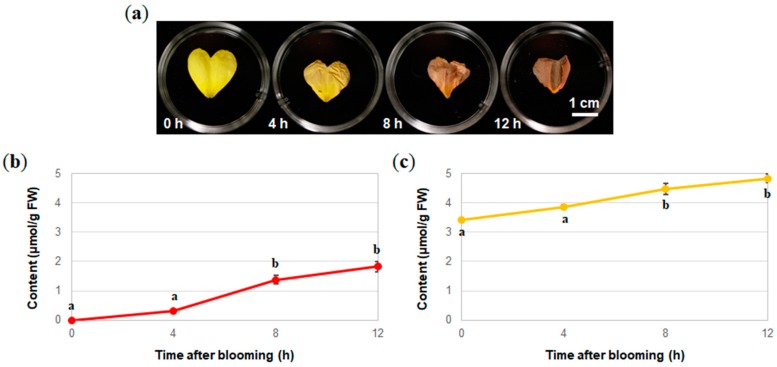
Changes in color and flavonoid components of the petals of *O. laciniata* during senescence. (**a**) Petal color at each stage, (**b**) change in the Cy3G (**1**) content, and (**c**) change in the isosalipurposide (**3**) content. The data shown are the means ± SE of three replicates (*n* = 3). Where no error bars are shown, the SE was too small to determine. Different letters indicate significant differences according to Tukey’s HSD test (*p* < 0.05).

Since the patterns of flavonoids in *O. laciniata* and *O. stricta* were almost the same, only the petals of *O. laciniata* were analyzed to determine the contents of Cy3G (**1**) and isosalipurposide (**3**) during senescence. The petals at 0 h, 4 h, 8 h, and 12 h after blooming were collected and extracted for HPLC analysis. The contents of both Cy3G (**1**) and isosalipurposide (**3**) were quantified over the course of 12 h after blooming (Figure 6). During senescence, the contents of both **1** and **3** increased with similar significant differences and the highest contents of the compounds were 1.82 µmol/g FW for Cy3G (**1**) and 4.83 µmol/g FW for isosalipurposide (**3**) 12 h after blooming (Figure 6).

## 3. Discussion

In this report, the flowers of *Oenothera* during the color change were chemically analyzed. In all three *Oenothera* species, Cy3G (**1**) was present in faded flowers, but no Cy3G was detected at full bloom (0 h). From the quantitative analysis of the flavonoids during flower senescence, increases in Cy3G (**1**) in both *O. tetraptera* and *O. laciniata* petals were observed. This corresponded to the color parameters and the UV/Vis absorption spectra. Therefore, it was concluded that the color change is due to the de novo synthesis of Cy3G.

Together with anthocyanin, we analyzed the flavonoid components and found that a high level of a flavonol known as quercitrin (**2**) was present in white petals of *O. tetraptera*. The molar ratio of **2** to **1** at 12 h after blooming was more than 13 to 1. The pH of the pressed juice of the *O. tetraptera* petals was approximately 5.5 (Table 1). At this pH, simple anthocyanins such as Cy3G are not stable and they are easily hydrated to give colorless pseudo bases. However, the high content of quercitrin (**2**) in the petals might stabilize the color of Cy3G by exhibiting a co-pigment effect. Yet, the yellow petals of *O. laciniata* and *O. stricta* contained glycosylchalcone and isosalipurposide (**3**). At 12 h, the molar ratio of **3** to **1** was approximately 2.5 to 1. These results correlated with previous reports on the flavonoid distribution in *Oenothera* [1,18,19,20]. In *O. laciniata* and *O. stricta*, the orange color in faded petals is developed by mixing yellow chalcone **3** with red Cy3G (**1**) [16,21,22]. In these petals, Cy3G might also be stabilized with isosalipurposide (**3**) and other co-existing polyphenolic compounds (Figure 5b).

According to the well-established flavonoid biosynthetic process [8,9,10,11,23], anthocyanins and flavonols are produced via a divergent pathway. Dihydroflavonol is the common precursor in the synthesis of both anthocyanidins by dihydroflavonol reductase (DFR) and flavonols by flavonol synthase (FLS) [10,11]. Since the content of **2** did not change during the experiment, **2** may not be involved in the Cy3G biosynthesis. In the case of chalcone **3**, its content increased in a similar way to the Cy3G content during *O. laciniata* flower senescence. The accumulation of chalcone **3** was reported to take place only due to the decrease in chalcone isomerase (CHI) activity, which catalyzes the conversion of chalcone into flavanone in the anthocyanin biosynthetic pathway [9,24]. Even though isosalipurposide (a chalcone glucoside) was proposed to have a different synthetic pathway from anthocyanin [8,9], chalcone is known to be an intermediate in anthocyanin biosynthesis [7,8,9,10,11,23,24]. This may suggest that, unless the biosynthesis of both **1** and **3** occurred during flowering, the synthetic pathways to **1** and **3** could converge. Further studies should be performed to clarify the relationship between the biosynthesis of flavonoids **1**–**3**. Studying the change in the color of *Oenothera* flowers can lead to understanding the mechanism of flower pigment synthesis during flowering. *Oenothera* might be an interesting model for further study into the Cy3G biosynthetic pathway.

## 4. Materials and Methods

### 4.1. Plant Materials

The *O. tetraptera* flowers used in this experiment were obtained from the Kochi Prefectural Makino Botanical Garden. The flower buds of *O. tetraptera* were cut, kept in a box, and transported to Nagoya University within 1 day. Afterward, flower buds were incubated in a plant growth chamber (14 h-light/10 h-dark cycle) at 25 °C (light) and 20 °C (dark) until sampling. The *O. laciniata* and *O. stricta* were grown at Nagoya University and the flowers in full bloom were collected and used for the experiment.

### 4.2. HPLC and Structural Analysis of the Flavonoids in the Petals

In both kinds of *Oenothera*, the blooming flowers were sampled at night (approximately 0 h to 2 h after blooming) while the senescent flowers were collected in the morning (approximately 12 h to 18 h after blooming). HPLC analysis was done on a small scale according to Yoshida et al. with some modifications [15]. Petals (1 mg) portion were extracted with 20 µL of 3% trifluoroacetic acid (TFA) in 50% aqueous acetonitrile (CH_3_CN). The extracts were analyzed by HPLC using a RPAQUEOUS-AR-3 column (2.0 × 150 mm) with linear gradient elution from 10% to 50% aqueous CH_3_CN containing 0.5% TFA.

To verify the structure of the flavonoids, LC-MS analysis was performed on a Bruker Daltonics micrOTOF-QII mass spectrophotometer with an Agilent 1200 Series HPLC system in an ESI-positive ion mode with the same HPLC conditions. The extracts were also co-chromatographed with authentic samples to confirm the structure of the compound.

### 4.3. Quantitative Analysis of the Flavonoids by HPLC

All the flower petals among flowers in full bloom were picked and weighed individually. After incubation in a growth chamber for the designated times (0 h, 4 h, 7 h, and 12 h for *O. tetraptera* and 0 h, 4 h, 8 h, and 12 h for *O. laciniata*), the petals were collected. The flavonoids in the petals were extracted and analyzed by HPLC, which was described above. The content of flavonoids was calculated using a standard curve prepared from the purified compounds [15]. The experiment was performed in triplicate. The obtained data were evaluated by one-way ANOVA using the post hoc Tukey’s HSD test (*p* values ≤ 0.05).

### 4.4. UV/Vis and Color Parameter Measurements

UV/Vis spectra as well as the color parameters of the *O. tetraptera* petals were measured by a JASCO V-560 UV/Vis spectrophotometer equipped with an integral sphere. The upper edges of the petals were cut into 1.5 × 1.5 cm squares for use in these analyses.

### 4.5. Petal pH Measurements

For petal pH measurements, fresh petals of *O. tetraptera* were ground and then the pH of the obtained petal juice was measured by a pH meter.

### 4.6. Isolation and Characterization of Isosalipurposide from Oenothera Laciniata

The petals of *O. laciniata* (150 g) were extracted twice with methanol. The crude extract was concentrated and dried under reduced pressure and the residue was re-suspended with 50% aqueous methanol. After sonification, the mixture was filtered through a PTEE membrane filter (pore size: 0.5 µm) and purified via preparative HPLC with an ODS-HG-5 column (25 mm i.d. × 250 mm) at a flow rate of 15 mL/min. The mobile phases were 0.1% TFA in 5% aqueous CH_3_CN for 0–5 min, 0.1% TFA in 20% aqueous CH_3_CN for 5–20 min, 0.1% TFA in 30% aqueous CH_3_CN for 20–35 min, and 0.1% TFA in 90% aqueous CH_3_CN for 35–50 min. The fractions were analyzed by HPLC to check their purity.

Pure isosalipurposide (**3**) was structurally analyzed by NMR, MS, UV/Vis, and IR techniques and used as a standard sample in the analysis of the flavonoids. ^1^H and ^13^C NMR spectra were obtained with a JEOL JNM-ECA-500 spectrometer. Chemical shifts are reported in ppm relative to CD_3_OD (δ = 3.31 ppm for ^1^H NMR and δ = 49.0 ppm for ^13^C NMR) as the reference. High-resolution mass spectra were recorded using a Bruker micrOTOF-QII electrospray ionization (ESI) spectrometer. IR spectra were obtained from KBr pellets on a JASCO FT/IR-460 plus spectrometer while UV/Vis spectra were collected by using a JASCO V-560 spectrophotometer (path length: 10 mm).

*Isosalipurposide* (*Chalconaringenin 2′-glucoside*), **3**: ESI-MS (positive mode) *m*/*z* = 435.1267 [M + H]^+^, λ_max_ (ethanol) = 371 nm (ε = 24,900). IR (ν_CO_) 1626 cm^−1^. ^1^H NMR (500 MHz, CD_3_OD) δ 8.02 (1H, d, *J* = 15.5 Hz; H-α), 7.67 (1H, d, *J* = 15.5 Hz, H-β), 7.61 (2H, d, *J* = 8.6 Hz, H-2, H-6), 6.83 (2H, d, *J* = 8.6 Hz, H-3, H-5), 6.22 (1H, d, *J* = 1.7 Hz, H-3′), 6.00 (1H, d, *J* = 2.3 Hz, H-5′), 5.14 (1H, d, *J* = 7.5 Hz, H-1″), 3.92 (1H, dd, *J* = 2.3, 12.6 Hz, H-6″_a_), 3.74 (1H, dd, *J* = 5.2, 12.0 Hz, H-6″_b_), 3.56 (1H, dd, *J* = 7.4, 9.2 Hz, H-2″), 3.51 (1H, t, *J* = 8.6 Hz, H-3″), 3.46 (1H, ddd, *J* = 2.3, 5.2, 12.6 Hz, H-5″), 3.44 (1H, t, *J* = 8.9 Hz, H-4″). ^13^C NMR (125 MHz, CD_3_OD) δ 194.5 (C=O), 167.8 (C-6′), 165.9 (C-4′), 161.8 (C-2′), 161.1 (C-4), 144.2 (C-β), 131.8 (C-2, C-6), 128.5 (C-1), 125.9 (C-α), 116.9 (C-3, C-5), 107.5 (C-1′), 101.9 (C-1″), 98.4 (C-5′), 95.7 (C-3′), 78.5 (C-3″, C5″), 75.0 (C-2″), 71.2 (C-4″), 62.4 (C-6″).

## Figures and Tables

**Table 1:** Color parameters and pH of *Oenothera tetraptera* petals during senescence.

Sample	CIELAB Color Coordinate ^1^			PH
	L*	a*	b*	
0 h	99.52	−1.5	3.33	5.73
4 h	93.13	8.5	−1.11	5.86
7 h	87.74	17.33	−3.94	5.66
12 h	82.39	19.62	−5.71	5.52

^1^ The Commission International de l’Eclairage (CIE) L* a* b* color parameters measure L*: lightness (0 = dark, 100 = bright); a*: green-red (negative = green, positive = red), and b*: blue-yellow (negative = blue, positive = yellow).

## Supplementary Materials

The following are available online, Figure S1: The LC-MS spectra of Cy3G (**1**), quercitrin (**2**) and isosalipurposide (**3**) from the extracts of *Oenothera* flowers, Figure S2: HPLC chromatogram of the extracts from petals of *O. stricta*, Figure S3: The ^1^H NMR spectrum (500 MHz) of isosalipurposide (**3**) in CD_3_OD at 25 °C, Figure S4: The ^13^C NMR spectrum (125 MHz) of isosalipurposide (**3**) in CD_3_OD at 25 °C, Figure S5: The COSY spectrum of isosalipurposide (**3**) in CD_3_OD at 25 °C, Figure S6: The NOESY spectrum of isosalipurposide (**3**) in CD_3_OD at 63 °C, Figure S7: The HMQC spectrum of isosalipurposide (**3**) in CD_3_OD at 25 °C, Figure S8: The HMBC spectrum of isosalipurposide (**3**) in CD_3_OD at 25 °C.
