# Supplementary material for: Change of Petals′ Color and Chemical Components in Oenothera Flowers during Senescence
Source: Molecules. 2018 Jul 12;23(7):1698. doi: 10.3390/molecules23071698 (PMC6099532; doi:10.3390/molecules23071698)
Supplement: Supplementary file 1 [file molecules-23-01698-s001.pdf]

Supporting Information

Mechanism of petal color change of *Oenothera* flowers during senescence by chemical analysis of flavonoid content

Yada Teppabut, Kin-ichi Oyama, Tadao Kondo and Kumi Yoshida

Supplementary materials

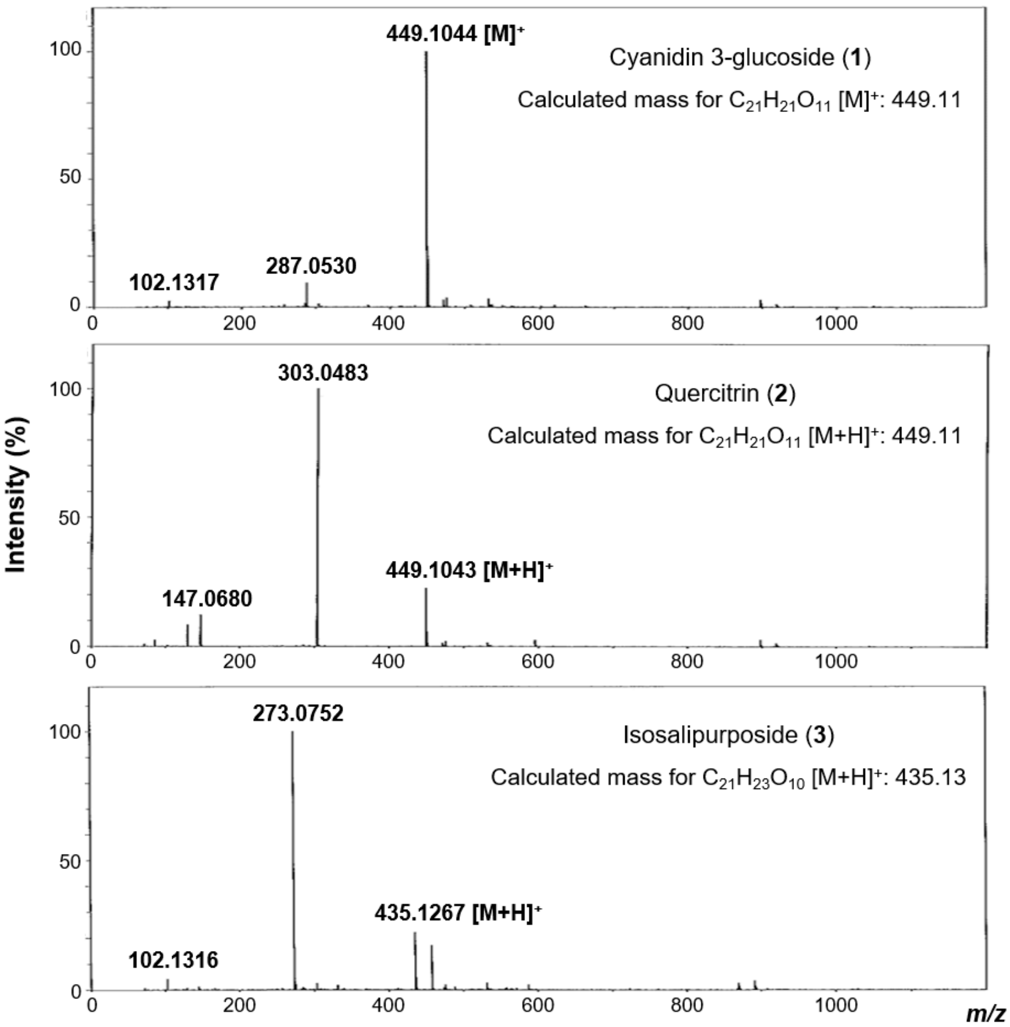

Figure S1. The LC-MS spectra of Cy3G (**1**), quercitrin (**2**) and isosalipurposide (**3**) from the extracts of *Oenothera* flowers. The spectra of Cy3G (**1**) and quercitrin (**2**) were obtained from the extract of *Oenothera tetraptera*, whereas the spectra of Cy3G (**1**) and isosalipurposide (**3**) was observed in *Oenothera laciniata* petal's extract.

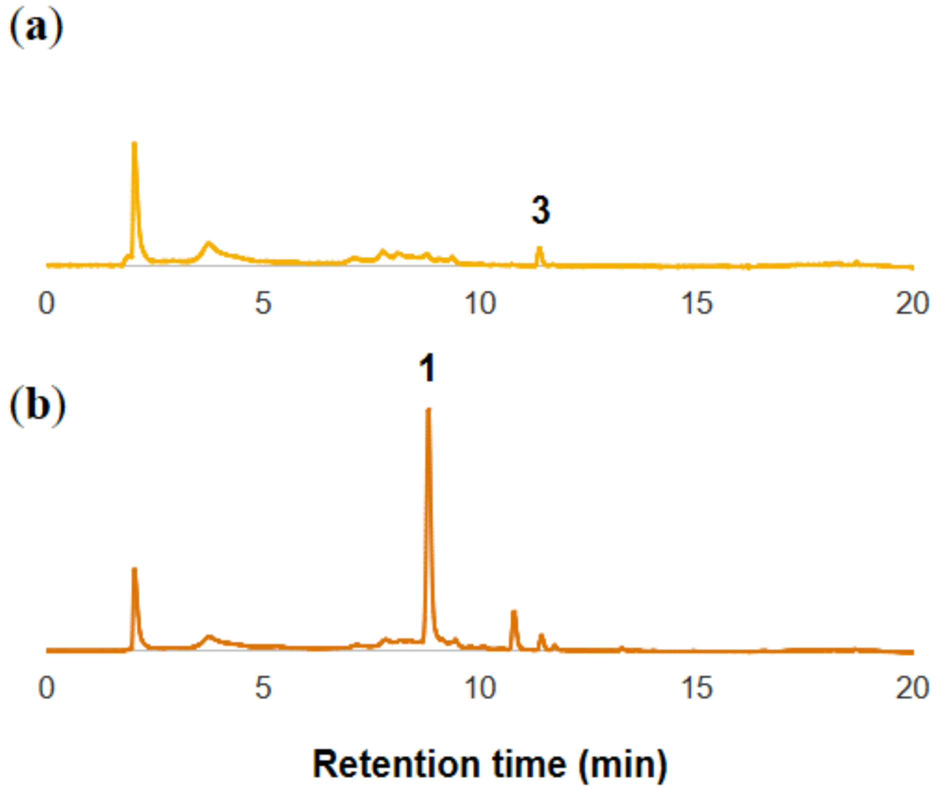

11 **Figure S2.** HPLC chromatogram of the extracts from petals of *O. stricta*. (**a**) Yellow petals at 0 h; (**b**)
12 Orange petals at 12 h.

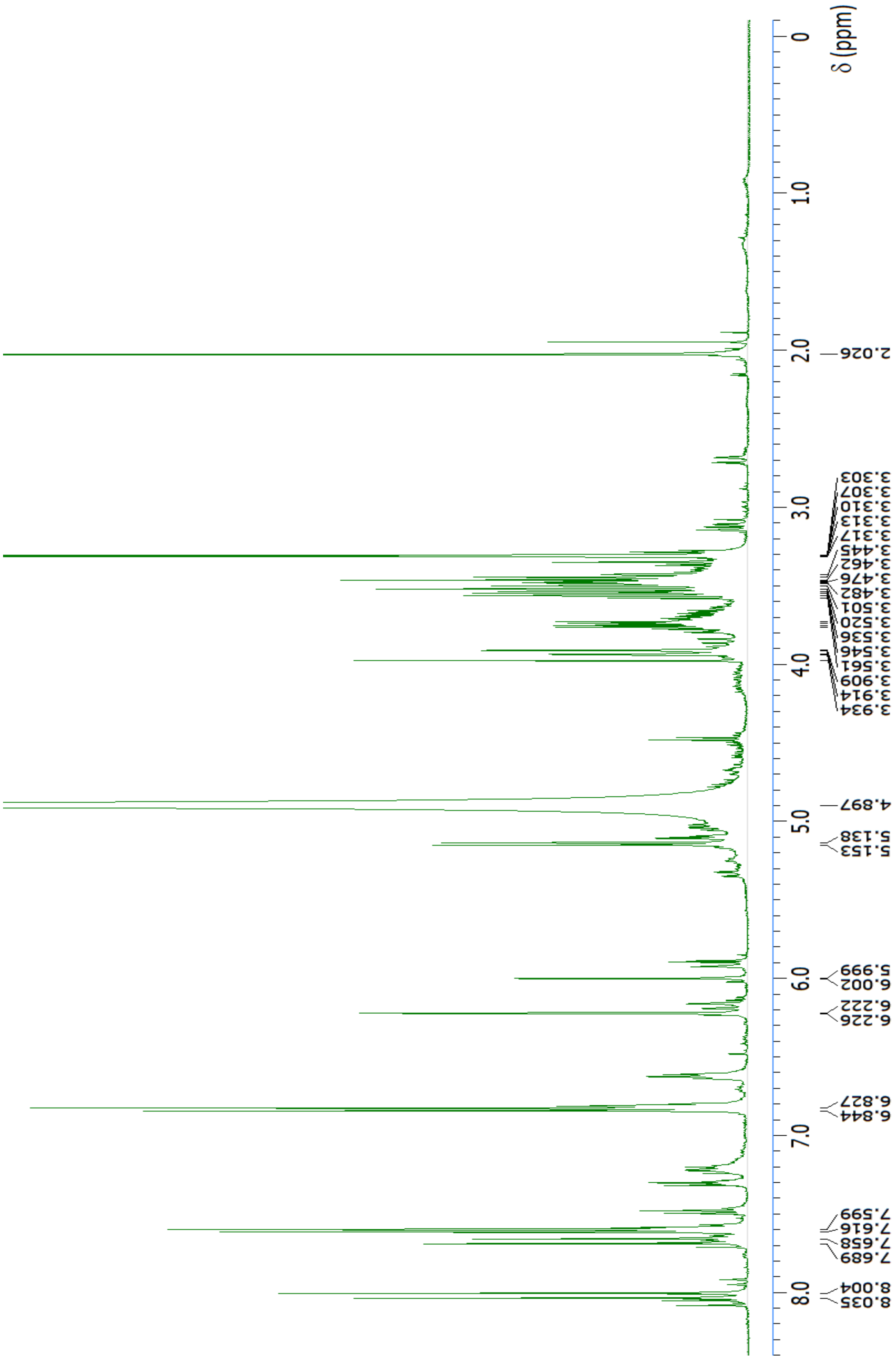

Figure S3. The ^{1}H NMR spectrum (500 MHz) of isosalipurposide (**3**) in CD_3OD at 25 °C.

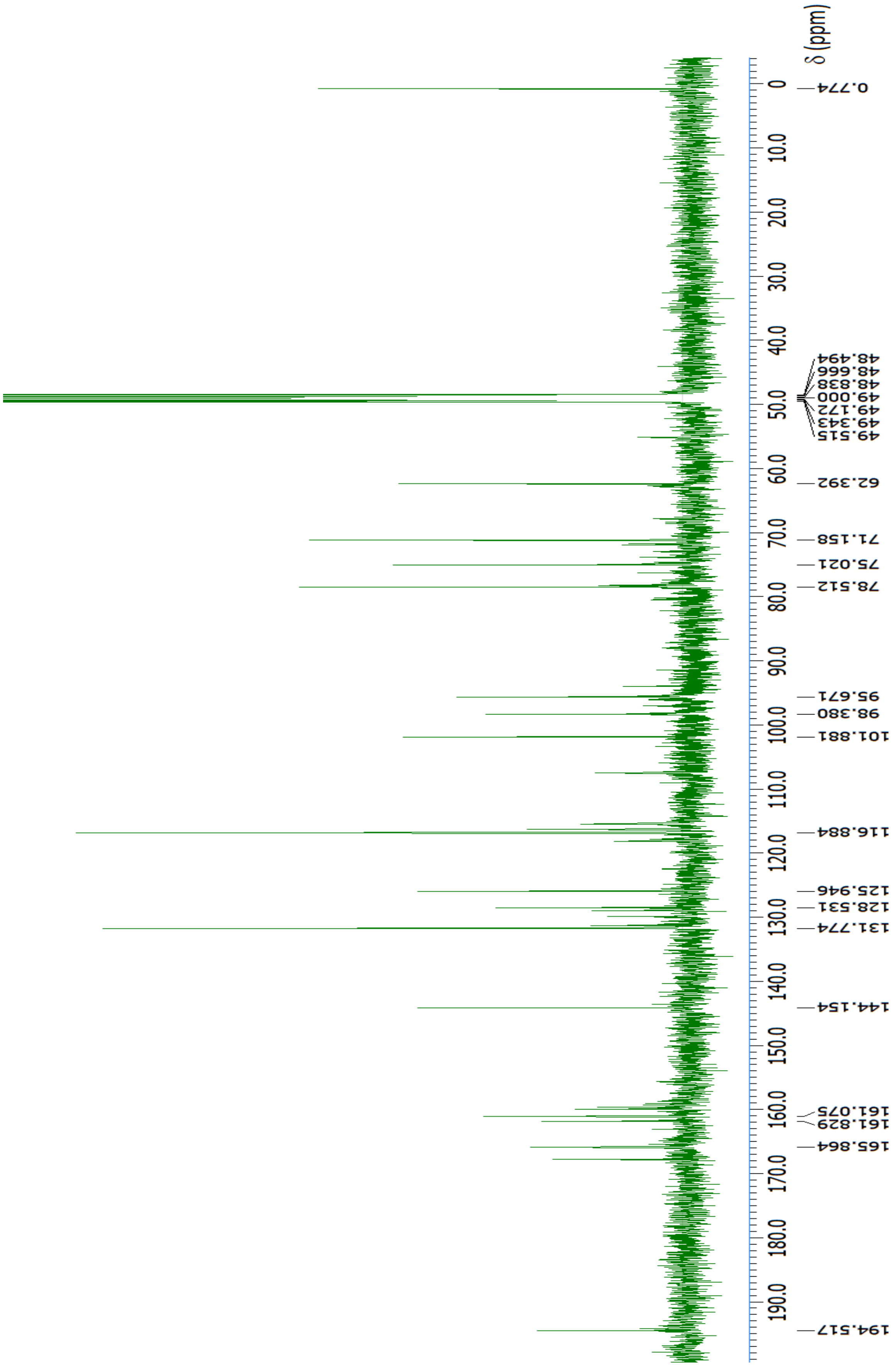

14 **Figure S4.** The ^{13}C NMR spectrum (125 MHz) of isosalipurposide (**3**) in CD_3OD at 25 °C.

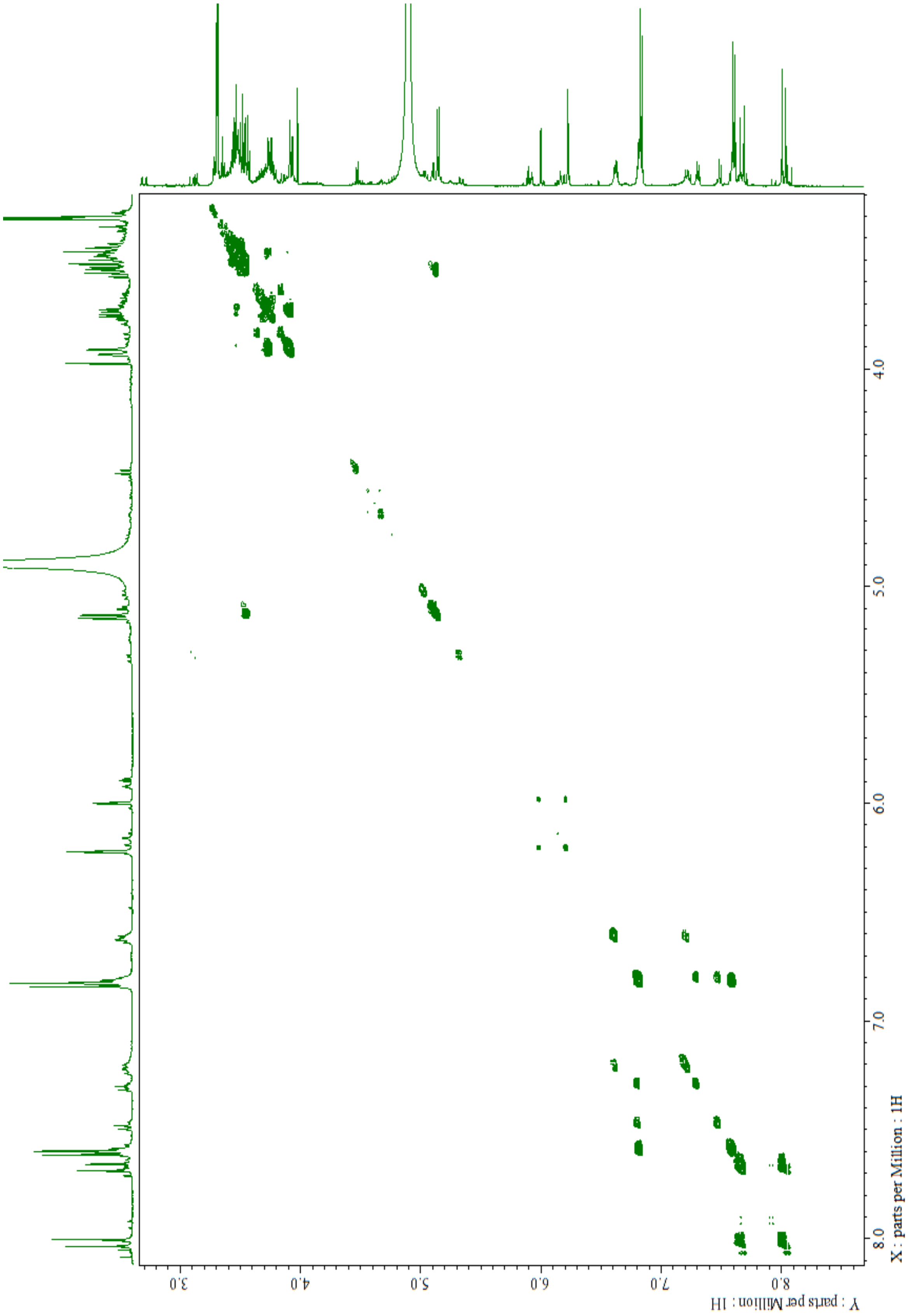

Figure S5. The COSY spectrum of isosalipurposide (**3**) in CD_3OD at 25 °C.

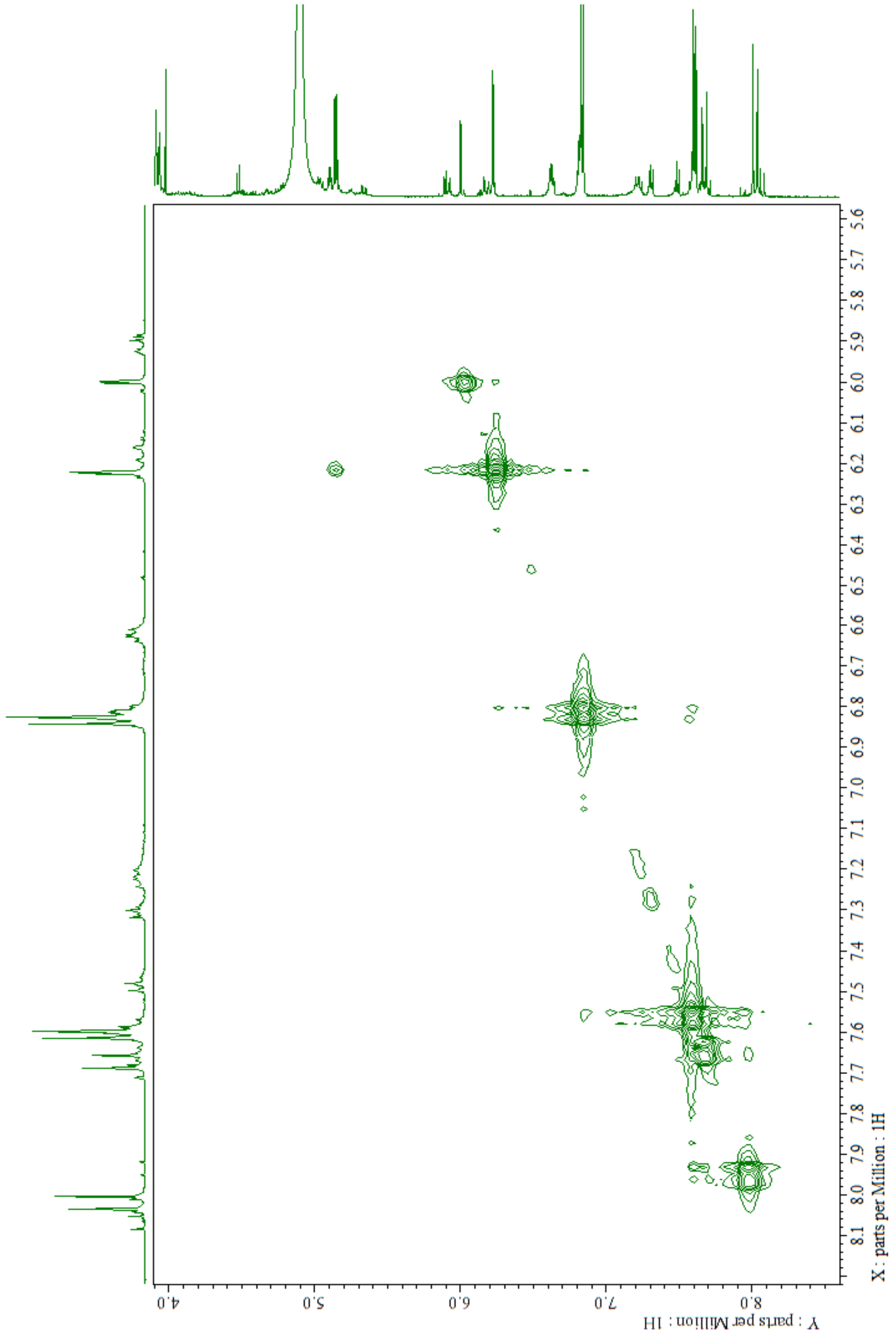

Figure S6. The NOESY spectrum of isosalipurposide (**3**) in CD_3OD at 63 °C.

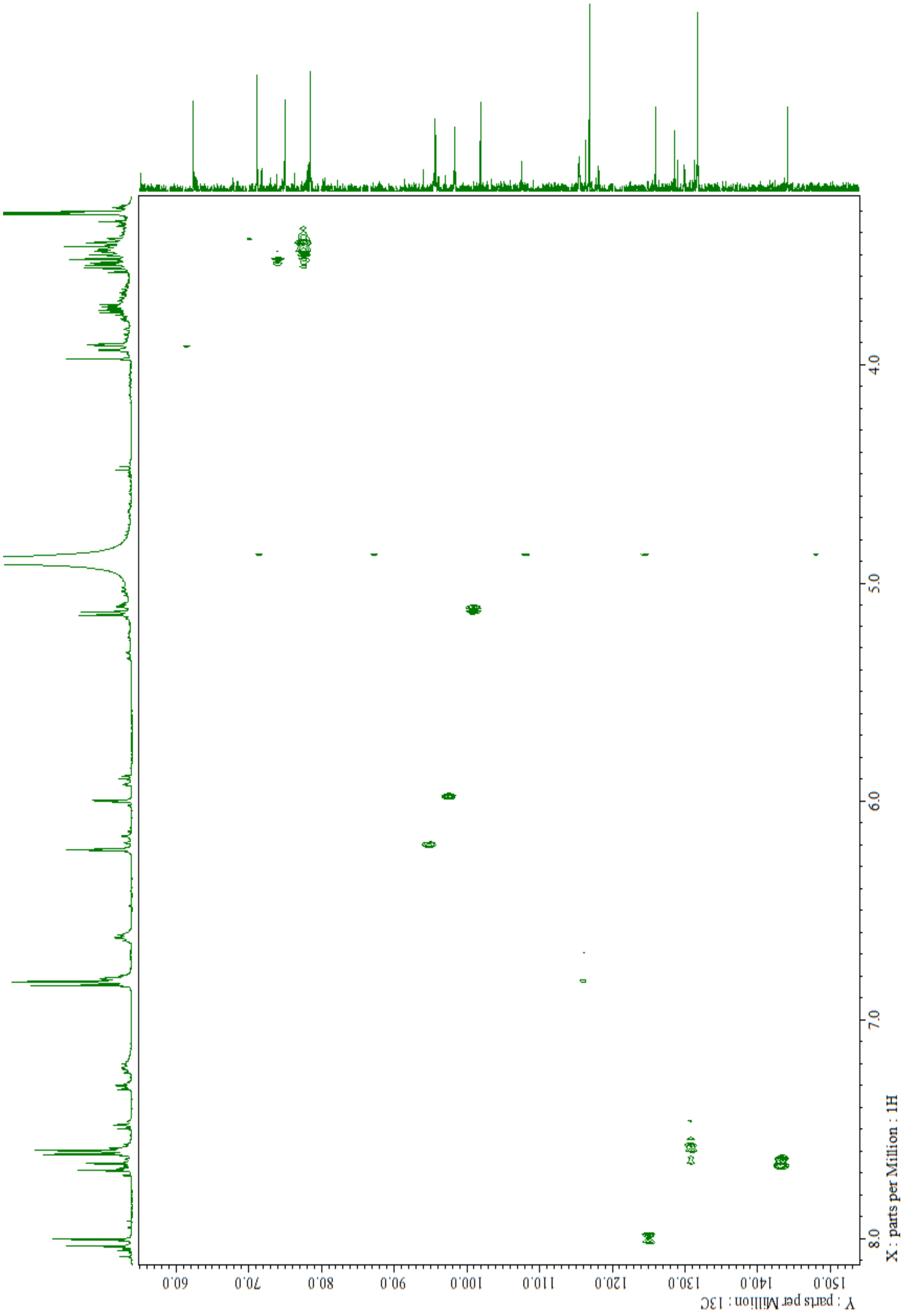

17 **Figure S7.** The HMQC spectrum of isosalipurposide (**3**) in CD_3OD at 25 °C.

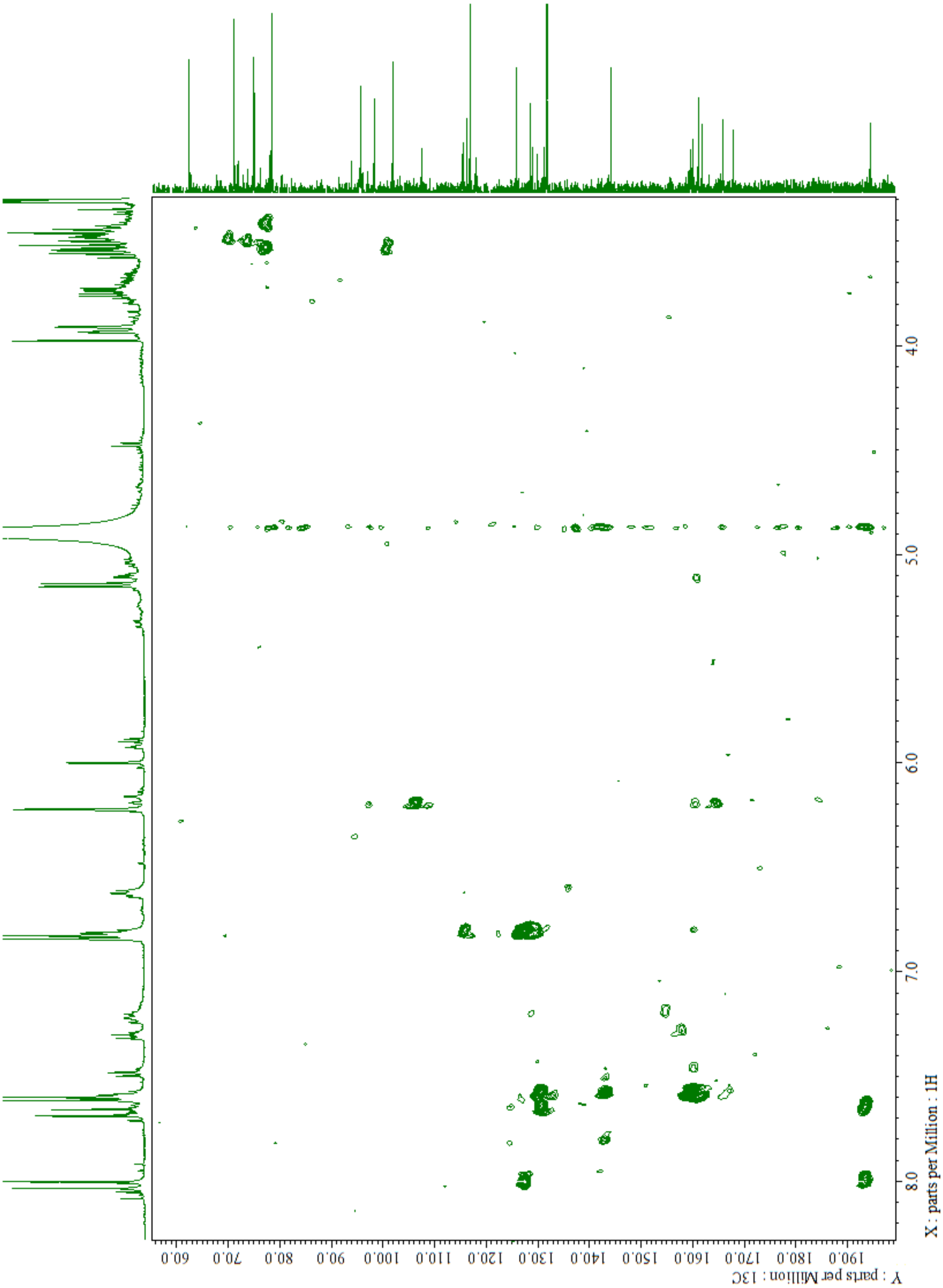

Figure S8. The HMBC spectrum of isosalipurposide (**3**) in CD_3OD at 25 °C.

© 2017 by the authors. Submitted for possible open access publication under the terms and conditions of the Creative Commons Attribution (CC BY) license (http://creativecommons.org/licenses/by/4.0/).
